# Immunization Campaigns and Strategies against Human Papillomavirus in Italy: The Results of a Survey to Regional and Local Health Units Representatives

**DOI:** 10.1155/2019/6764154

**Published:** 2019-07-04

**Authors:** Cecilia Trucchi, Claudio Costantino, Vincenzo Restivo, Chiara Bertoncello, Francesca Fortunato, Silvio Tafuri, Daniela Amicizia, Domenico Martinelli, Chiara Paganino, Maria Francesca Piazza, Federico Tassinari, Valentino Tisa, Pasquale Stefanizzi, Vincenzo Baldo, Alessandra Casuccio, Rosa Prato, Filippo Ansaldi, Giancarlo Icardi

**Affiliations:** ^1^Azienda Ligure Sanitaria della Regione Liguria (A.Li.Sa.), Liguria Region, Italy; ^2^Policlinic San Martino Hospital, Genoa, Italy; ^3^Department of Science for Health Promotion and Mother to Child Care “G. D'Alessandro”, University of Palermo, Italy; ^4^Department of Cardiac, Thoracic and Vascular Sciences, University of Padua, Italy; ^5^Department of Medical and Surgical Sciences, University of Foggia, Italy; ^6^Department of Biomedical Science and Human Oncology, University of Bari, Italy; ^7^Department of Health Sciences, University of Genoa, Italy

## Abstract

*Objective. *The study aimed to assess the impact of HPV immunization campaigns organizational aspects, the characteristics of immunization program (vaccination targets and type of offer), and communicative strategies adopted by four Italian administrative regions on vaccination coverage observed.* Methods. *From November 2017 to March 2018, regional and Local Health Units (LHUs) representatives were invited to complete an online survey including 54 questions evaluating vaccination invite systems, access systems to vaccination centres, reminder and recall systems, and adverse events surveillance. An overall descriptive analysis was conducted. Since observed vaccine coverage (VC) obtained in females (2002-2004 birth cohorts) was lower than objectives fixed by the Italian Ministry of Health, variables were assessed using the national VC mean obtained in the 2003 girls birth cohort as outcome.* Results. *Twenty-six LHUs belonging to 4 Northern and Southern Italian regions participated in the study. Organizational aspects significantly related to VC lower than the national mean were access to vaccine centres without appointment and parents' reservation as appointment planning system. Recall systems for both the first and the second dose, including the appointment in the invitation letter, the availability of regional immunization registry, and education of healthcare workers on universal HPV immunization strategies, instead, were related to higher VC. As regards preadolescent immunization strategies, both VC obtained in girls and boys were far from the Ministerial goals. Only 20% of LHUs introduced multicohort female strategies while all LHUs adopted copayment targeting both men and women. Immunizations strategies targeting subjects at risk were implemented only in half of participating LHUs.* Conclusions. *VC observed in participating LHUs are largely lower than the national objectives in all anti-HPV vaccine targets. Both organizational and educational strategies have to be implemented to improve the VC goals.

## 1. Introduction

Human papillomavirus (HPV) is considered one of the most common sexually transmitted infections in the world, affecting people especially in developed countries. About 70-80% of the sexually active women and men will acquire an HPV infection during their life [1]. Since HPV was discovered to cause cervical, penile, anal, vaginal, oropharynx, hypopharynx, and larynx cancers, the development of effective anti-HPV vaccines was considered one of the most important public health goals to be achieved [2–4]. In 2006, the European Medicines Agency (EMEA) approved a quadrivalent vaccine containing antigens against four HPV-types (6, 11, 16, and 18) and, one year later, they approved a second bivalent vaccine against oncogenic HPV-types (16 and 18). Finally, in 2015, a nonavalent vaccine extending antigens to five additional HPV-types (31, 33, 45, 52, and 58) to that contained in the quadrivalent vaccine was approved by EMA [5, 6].

Initially, HPV vaccination has been recommended for adolescent women prior to sexual debut, and it has already been showed to be highly effective in reducing HPV-related lesions, such as genital warts as well as Cervical Intraepithelial Neoplasia (CIN) [7]. Increasing evidence demonstrated the consistent burden of HPV-related diseases also in men, such as anal, head, and neck cancers (4 times or more than women) and penile cancers [8, 9].

Nowadays USA, Canada, Australia, and over 37 out of 53 European (EU) countries have introduced the vaccine into their national routine immunization schedules and several of these both for males and females [10].

In 2014, HPV vaccination coverage in the EU Region accounted for about 50% in the primary target, while African countries reported a mean 88% HPV vaccination coverage (VC) [11]. In Italy, HPV vaccination is free and has been actively offered to all girls during their 12th year of life since 2007, and recently the National Vaccination Plan (PNPV) 2017-2019 established a target vaccination coverage of 95%, within three years of the start of the campaigns [12]. However, despite several promotional activities, VC is largely unsatisfactory, ranging from 30% to 75% among Italian administrative regions for full HPV vaccination in primary cohorts (birth cohort: 2005) of women and from 0% to 65% among primary cohorts of men [13].

In Europe, several studies have previously indicated that common reasons for not receiving or completing the HPV vaccine were the perception of low risk or not needing the vaccine, low perception of HPV vaccination benefits, doubt about the safety and efficacy of the vaccines, fear of side effects, low participation at school seminar on HPV for school-based immunization campaign, lack of physician recommendations, and cost of the vaccine [14–17].

Organizational aspects to improve HPV vaccine uptake were also investigated, in particular the implementation of immunization services' accessibility, the role of reminder systems, and communication technologies. Nevertheless, previous evaluations were rarely comprehensive or specific, thus compromising the estimate of the effect of interventions' interaction and the consideration of HPV vaccine peculiarities (i.e., the variety of involved targets and healthcare professionals, the role of parents, and the range and seriousness of HPV-related diseases, including cancers) [18–29].

Aim of the present study was assessing the different HPV immunization campaigns carried out in four Italian administrative regions, evaluating the impact of organizational aspects, the characteristics of immunization program (vaccination targets, e.g., female, male, categories at risk such as men who have sex with men, etc., and type of offer), and communicative strategies adopted by the regions on vaccination coverage observed.

## 2. Material and Methods

An online questionnaire was administered through Google Drive platform to 26 Local Health Units (LHUs), belonging to 4 Northern and Southern Italian regions, where the universal preadolescents HPV vaccine recommendation was introduced before the PNPV 2017-2019, covering 25.7% of the national population.

From November 2017 to March 2018, regional and LHUs representatives were invited to complete an online survey including 54 questions evaluating HPV immunization policies targeting preadolescents, adults and subjects at risk, obtained results in different targets, communication and education strategies, and organizational characteristics of vaccination centres. In particular, vaccination invite systems, subjects who invites preadolescents, sending information with invitation letter, subjects who administers the vaccine, access system to vaccination centres, appointment planning system, reminder of the first dose appointment, specific HPV vaccine sessions, recall of subjects who missed the first and the second dose, second dose appointment planning, and AE surveillance within 30 minutes and since the day after were investigated. An overall descriptive analysis was conducted.

Since observed VC obtained in females 2002-2004 birth cohorts were lower than the objectives fixed by the Ministry of Health, VC national mean was used as outcome. In particular, the possible association of organizational aspects with VC obtained in the female 2003 birth cohort higher than the national mean (64.7%) [13] was tested through univariable logistic regression.

VC obtained in boys were not considered as outcome since the objectives fixed by the Ministry of Health in PNPV 2017-2019 refer to the 2006 birth cohort for which data were not available.

After the collinearity assessment, factors resulting statistically significant in univariable comparisons (p < 0.05) were included in a multivariable model, by means of a stepwise backward procedure.

Statistical analyses were conducted by the JMP software, version 13.

### 2.1. Ethical Approval

The study protocol was approved by the Regional Ethic Committee of the Liguria region, Italy (P.R. 162REG2017).

## 3. Results

### 3.1. Universal Preadolescents HPV Vaccination Organizational Aspects

The main results concerning universal preadolescents HPV vaccination organizational aspects are shown in Table 1. Almost all LHUs invite preadolescents through letters addressed to parents, which in 76.9% of cases included also informative material. Schools are involved only in 26.9% of cases. The access system to vaccination centre for the first dose administration was by appointment in almost all LHUs, and the appointment was included in the invitation letter in 76% of cases. The second dose appointment was planned during the first appointment in 80.8% of cases. Only about half of the LHUs adopt reminder systems for the first dose appointment. HPV vaccine is administered in specific vaccine sessions in 80.8% of LHUs and recall systems for subjects who missed the first and the second dose are used in 69.2% and 61.5% of cases, respectively. As regards adverse events surveillance after the HPV vaccine administration, about 20% of LHUs reported no surveillance within 30 minutes and no active regional vaccine-vigilance system.

### 3.2. Immunization Strategies and Vaccine Coverage

**Figure 1:**
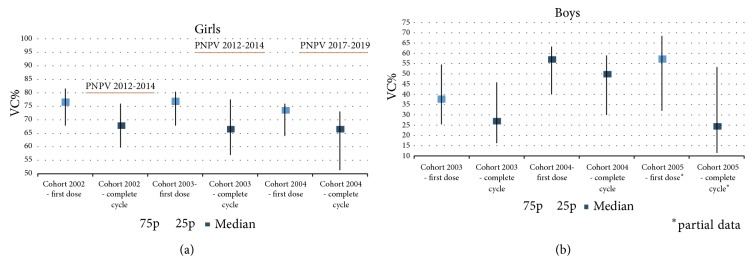
Vaccine coverage (median, 25-75 p) for first dose and complete cycle obtained in preadolescents girls (birth cohorts 2002-2004) (a) and boys (birth cohorts 2003-2005) (b) in participating Local Health Units and vaccine coverage objectives set by the Italian Ministry of Health.

As regards preadolescent immunization strategies, both VC obtained in boys and girls are largely suboptimal in participating LHUs and very far from the goals set by the Ministry of Health in the PNPV 2012-2014 and 2017-2019 (Figures 1(a) and 1(b)). In particular, median VC for complete cycle obtained in 2002, 2003, and 2004 girls birth cohorts were 67.7% (25-75p=59.7%-75.9%), 66.5% (25-75p=57%-77.5%), and 66.5% (25-75p=51.4%-73.1%), respectively. In boys the VC for complete cycle were 26.8% (25-75p=16.3%-45.8%) and 49.7% (25-75p=59.7%-75.9%) in 2003 and 2004 birth cohorts, respectively.

Furthermore, only about 20% of LHUs introduced multicohort female strategies while all adopted copayment targeting both men and women.

Immunizations strategies targeting subjects at risk are implemented only in half of participating LHUs and a multidisciplinary network to identify them is active in 27% of cases. The healthcare workers who recommend the HPV vaccine are various but low in numbers (immunization centres, gynaecologists, infectious diseases specialists, and general practitioners in 41.7%, 25%, 33.4%, and 25%, respectively), while the administration of HPV vaccine is centralized in vaccine centres.

As regards vaccine registries, they are digitized in all participating LHUs but only about the half of them are present on a regional level.

### 3.3. Communication Strategies

Communication tools were also investigated (Table 2). In particular, informative material available at the immunization centres is prepared locally in the majority of cases and translated in other languages than Italian only in 19.2% of cases. A call centre to discuss about vaccines is active in 53.8% of cases, formative moments such as focus group addressed to preadolescents parents were conducted in 42.3% of cases, and local media were involved in informative campaigns in about 35% of LHUs. The education of healthcare workers on universal strategy was multidisciplinary and it was conducted in almost all the LHUs. The analysis of suboptimal obtained VC and of vaccine hesitancy determinants was conducted only in 57.7% and 34.6% of cases, respectively.

Table 3 shows the results of univariable logistic regression investigating the possible association between organizational aspects and VC obtained in the female 2003-birth cohort. Organizational aspects significantly related to VC lower than the national mean were access to vaccine centres without appointment and parents' reservation as appointment planning system.

Recall systems for both the first and the second dose, including the appointment in the invitation letter, the availability of regional immunization registry, and education of healthcare workers on universal HPV immunization strategies, instead, were related to higher VC.

After multivariable analysis, one variable resulted statistically significant: access without appointment to vaccination centres (p=0.038); recall systems resulted borderline, with p=0.063 (Table 4). In particular, the probability to obtain VC higher than the national mean is equal to 87.6% if a recall of subjects missing the second dose is active. If the access system to vaccine centre is without appointment, the probability to obtain VC higher than the national mean is only 8.7%. The two organizational aspects are related to a probability of gaining higher VC of about 37%.

## 4. Discussion

This survey allowed us to obtain a detailed picture of a wide range of HPV vaccine offer and promotion strategies and to identify actions adopted in Italian LHUs that are associated with VC higher than the national mean.

In our knowledge, the most studied strategies aimed at gaining adequate HPV VC deal with the evaluation of knowledge, attitudes, and determinants of acceptance and refusal, and they mainly focus on preadolescents girls and their parents [30–41].

Nevertheless, immunization strategies and organizational aspects of vaccine centres also have a relevant role in determining the vaccine compliance.

Some studies investigated interventions to improve HPV vaccine uptake but each of them was evaluated separately [18–23]. Other studies and recommendations on factors influencing VC integrated the evaluation of many interventions but rarely specific for HPV vaccine, that is critical for the variety of targets and healthcare professionals implicated in vaccine counselling, the involvement of parents, and the relevance of indications including cancers [24–27]. In particular, the Community Preventive Services Task Force (CPSTF) supported by the Centers for Disease Control and Prevention (CDC) provides evidence-based findings and recommendations on intervention approaches for increasing vaccination, based on available scientific evidences [28]. Findings are divided into three categories [29], including recommended with strong evidence strategies aiming at enhancing access to vaccination services (e.g., home visits, vaccination programs in schools and organized child care centres, and vaccination programs in women, infants, and children settings), increasing community demand for vaccinations (e.g., client reminder and recall system and vaccination requirements for child care, school, and college attendance), and provider- or system-based interventions (e.g., immunization information systems, provider assessment and feedback, and provider reminders).

Among the abovementioned interventions, the active invitation of eligible subjects represents one of the more effective interventions to increase the VC. In this context, almost all participating LHUs invite adolescents' parents by letter and adopt the appointment as access system to vaccination centres, limiting the free access to 19.2% of cases. In particular, the appointment is included in the invitation letter in 76% of cases.

On the contrary, the access to vaccine centres without appointment and parents' reservation as appointment planning system are significantly related to VC lower than the national mean. Further, recall systems for both the first and the second dose is adopted only in 69.2% and 61.5% of cases, even if recommended with strong evidence by scientific authorities [29] and resulted significantly related to higher VC also in our study.

Among recommended with strong evidence immunization information systems, the digitized vaccine registries could be considered. Even if they are active in all participating LHUs, only about the half reported regional immunization strategies, that resulted significantly related to higher VC. This limits the sharing of immunization data between LHUs and from LHUs to regional authorities, compromising the governance of immunization policies.

Even though adverse events surveillance could not be strictly considered an organizational aspect, we investigated routine system used by LHUs to monitor adverse events as relevant quality and safety standard of immunization policies. Surprisingly, 30 minutes surveillance and regional vaccine-vigilance systems are not conducted in all participating LHUs and thus they should be extended and homogenised.

As regards communication strategies, almost all participating LHUs reported education activities on universal HPV immunization strategy targeting health care workers. Education targets the main professionals involved in HPV vaccine counselling but the proportions are low and the interventions are conducted periodically only in the half of LHUs, highlighting the need of promoting further activities. This is of particular relevance in the field of HPV primary and secondary prevention since a variety of professionals operating in different healthcare settings are involved along a decision path where the immunization centre often represent the point of arrival. Furthermore, available evidences demonstrate the importance of the role of trusted healthcare provider in taking decisions in the field of immunization [25, 26, 33, 40–42]. In particular, the HPV vaccine targets preadolescents of both sexes and subjects at risk; thus paediatricians and general practitioners ease the link between subjects and immunization centre. Parents are also involved in the decision path and they usually identify gynaecologists as reference figure to obtain information on a sexually transmitted infection causing anogenital cancers. Subjects at risk could refer to infectious disease specialists and oncologists but also dermatologists, urologists, and otolaryngology specialists. Thus, the synergy between professionals and their active role in vaccine offer are essential to obtain a good compliance to HPV prevention strategies, including vaccines.

Furthermore, sharing a unique and coherent message among different stakeholders using available communication tools is crucial to obtain the best results in terms of adherence to HPV vaccine. As healthcare planning activities should include the phase of feedback and report, the discussion of obtained VC and the analysis of vaccine hesitancy determinants among healthcare workers were also investigated, showing a prevalence as low as 57.7% and 34.6%, respectively.

The studied communications strategies directed to target subjects included the availability of informative material at immunization centres that was prepared by LHUs and regions in 50% and 42.3% of cases, respectively, and translated in other languages only in 19.2% of cases. Even if these factors are not significantly related to VC, they could contribute to the circulation of scientifically correct information and compensate the spread of misleading messages by “no-vax”. Furthermore, call centres coordinated by immunization centres to obtain information and address doubt about vaccines are available in about the half of participating LHUs. The involvement of media and the conduction of focus group in particular in the school setting could increase the vaccine demand by target when combined with other activities; nevertheless, they are reported in less than half of participating LHUs. This could be due to the suboptimal economic and human resources currently available for prevention in the majority of Italian regions.

Our findings are in line with the most recent evidences on interventions aimed at improving HPV vaccine uptake [42–46]. In particular, recall systems for missed administrations and free access to vaccination centres resulted in the main factors related to VC outcome in preadolescent girls, demonstrating the importance of implementing the accessibility to immunization services and the taking-care process of target subjects by health care professionals.

Of concerns, no participating LHUs gained VC objectives fixed by the Italian Ministry of Health, not only for the recent target represented by preadolescent boys but also for the more consolidated girls target. Furthermore, even if the PNPV 2017-2019 recommend the immunization of male who have sex with men and some regions correctly included among subjects at risk the HIV positive, the implementation of specific strategies is largely suboptimal. These observations are particularly serious from a public health perspective, given the high prevalence of HPV infection and the variety and burden of HPV-related diseases [1–4].

The strengths of our study are represented by the wide spectrum of organizational aspects and communication tools evaluated and the estimate of their role in determining the best outcome in terms of VC. In Italy, from 2010 to 2013 the study “local and evaluation of HPV immunization campaigns against HPV, VALORE” was coordinated by the Ministry of Health and by the* Istituto Superiore di Sanità* in order to improve the compliance to HPV vaccine and provide the regional and LHUs authorities the operational tools to increase VC [47]. Various organizational aspects and communications activities were investigated; nevertheless the boys did not represent the target of HPV vaccine during the study period and no multivariable analysis was conducted to consider diverse independent variables simultaneously.

The main limitations of the study are the difficulty in quantifying and exhaustively evaluating all organizational aspects of immunization centres and communication strategies and the number of participating LHUs that could compromise the representativeness of the national picture. Nevertheless, participating LHUs belong to four among the most populous Italian regions, with heterogeneous sociodemographic characteristics and healthcare systems. In particular, school-based programs were not investigating, even if robust evidences demonstrate their role in increasing the compliance to HPV vaccine. Nevertheless, the paucity of human resources in immunization centres reported in a wide proportion of participating LHUs limits the feasibility of this strategy.

## 5. Conclusions

In conclusion, our study demonstrated that the majority of Italian LHUs implemented proved actions (e.g., active free offer, invite letter, and recall of subjects who missed vaccine administration) concurrently with some HPV vaccine promotion and communication strategies directed to target subjects and healthcare workers. Nevertheless, VC observed in participating LHUs are largely lower than the national objectives in all HPV vaccine targets and organizational strategies to reach subjects at risk are suboptimal.

Since multicomponent interventions have a synergistic effect, both organizational and educational strategies have to be implemented to improve HPV VC.

## Figures and Tables

**Table 1:** Universal pre-adolescents HPV vaccination organizational aspects.

Organizational aspects	(N=26)
	N (%)
Vaccination invite system	
*Letter addressed to parents by LHUs*	25 (96.2%)
*Information given at school*	7 (26.9%)
*SMS to parents*	1 (3.8%)
*Smartphone Application*	1 (3.8%)

Subject who invites pre-adolescents	
*Healthcare workers of immunization centres*	26 (100%)

Sending information with invitation letter	20 (76.9%)

Subjects who administers the vaccine	
*Healthcare workers of immunization centres*	26 (100%)

Access system to vaccination centres	
*Free access*	5 (19.2%)
*Appointment*	25 (96.1%)

Appointment planning system	
*Included in the invitation letter*	19 (76%)
*Parents reservation*	10 (38.5%)

Reminder of the first dose appointment	14 (53.4%)

Specific HPV vaccine sessions	21 (80.8%)

Recall of subjects who missed the first dose	18 (69.2%)
*Letter*	14 (77.8%)
*Phone call*	9 (50.0%)
*SMS*	1 (5.9%)

Second dose appointment planning	
*During the first dose appointment*	21 (80.8%)
*Invitation letter*	4 (15.4%)
*Parents reservation*	1 (3.8%)

Recall of subjects missing the second dose	16 (61.5%)
*Phone call*	8 (50%)
*Invitation letter*	10 (62.5%)
*SMS*	1 (6.3%)

AE surveillance within 30 minutes	21 (80.8%)

AE surveillance since the day after	
*Parents contact to immunization centre*	17 (65.4%)
*Report*	9 (34.6%)
*Regional vaccine-vigilance system*	21 (80.8%)

LHU=Local Health Unit; SMS=Short Message Service; AE=Adverse Event

**Table 2:** Communication strategies about HPV vaccine.

Communication strategies	(N=26)
	N (%)
Informative material available at immunization centres	
*Ministry of Health*	2 (7.7%)
*Region*	11 (42.3%)
*LHUs*	13 (50%)
*Scientific agencies*	3 (11.5%)
*Pharmaceutical companies*	8 (30.8%)

Translation of informative material	5 (19.2%)

Call center	14 (53.8%)

Focus group	11 (42.3%)

Media	9 (34.6%)

Education of HCWs on “universal” strategy	24 (92.3%)
*Immunization centres*	16 (66.7%)
*General practitioners/Pediatricians*	14 (58.3%)
*Gynecologists*	9 (37.5%)
*Screening centres*	9 (37.5%)
*Periodically*	12 (50%)

Discussion of obtained vaccine coverages	15 (57.7%)

Analysis of vaccine hesitancy determinants	9 (34.6%)

**Table 3:** Univariable logistic regression of universal pre-adolescents HPV-vaccination vaccination organizational aspects associated with gaining by LHU of vaccine coverage in 2003 girls birth cohort higher than the national mean.

Organizational aspects and communication strategies	*Cohort 2003*			
	*LHU VC> National mean VC (64.7%)*			
	Yes	No	P-value	OR (95% C.I.)
Access system to vaccination centres				
*Free access*	1 (5.9%)	4 (44.4%)	0.018	0.08 (0.01-0.87)

Recall of subjects who missed the first dose	14 (82.4%)	4 (44.4%)	0.046	5.8 (1-35.7)

Recall of subjects missing the second dose	13 (76.5%)	3 (33.3%)	0.032	6.5 (1.09-38.63)

Appointment planning system				
*Included in the invitation letter*	15 (88.2%)	4 (44.4%)	0.017	9.37 (1.3-67.65)
*Parents reservation*	1 (5.9%)	4 (44.4%)	0.018	0.08 (0.01-0.87)

Immunization registry				
*Regional *	12 (70.6%)	2 (22.2%)	0.019	8.4 (1.27-55.39)

Education of HCWs on “universal” strategy	17 (100%)	7 (77.8%)	0.043	NA

LHU=Local Health Unit; VC=Vaccine Coverage; HCW=Health Care Worker

**Table 4:** Organizational aspects selected by multivariate stepwise logistic regression for prediction of gaining by LHU of vaccine coverage in 2003 girls birth cohort higher than the national mean.

Organizational aspects		Recall of subjects missing the second dose (p=0.063)	
		Yes	No
Free access to vaccination centres (p=0.038)	Yes	36.9%	8.7%
	No	87.6%	53.4%

## Data Availability

The data used to support the findings of this study are available from the corresponding author upon request.

## References

[1] Xavier B. F., de Sanjosé S. (2007). The epidemiology of human papillomavirus infection and cervical cancer. *Disease Markers*.

[2] Kahn J. A., Burk R. D. (2007). Papillomavirus vaccines in perspective. *The Lancet*.

[3] zur Hausen H. (2009). The search for infectious causes of human cancers: where and why. *Virology*.

[4] Hartwig S., Syrjänen S., Dominiak-Felden G., Brotons M., Castellsagué X. (2012). Estimation of the epidemiological burden of human papillomavirus-related cancers and non-malignant diseases in men in Europe: a review. *BMC Cancer*.

[5] Pharmacovigilance Risk Assessment Committee (PRAC) (2019). *Assessment Report. Review under Article 20 of Regulation (EC) No 726/2004*.

[6] Van Dammea P., Bonanni P., Boschc F. X. (2016). Use of the nonavalent HPV vaccine in individuals previously fully or partially vaccinated with bivalent or quadrivalent HPV vaccines. *Vaccine*.

[7] Brotherton J. M., Fridman M., May C. L., Chappell G., Saville A. M., Gertig D. M. (2011). Early effect of the HPV vaccination programme on cervical abnormalities in Victoria, Australia: an ecological study. *The Lancet*.

[8] Ramqvist T., Dalianis T. (2010). Oropharyngeal cancer epidemic and human papillomavirus. *Emerging Infectious Diseases*.

[9] Ong J. J., Walker S., Grulich A. (2019). Incidence, clearance, and persistence of anal human papillomavirus in men who have sex with men living with human immunodeficiency virus. *Sexually Transmitted Diseases*.

[10] World Health Organization Europe (2019). *HPV Vaccination: Protecting Girls Now from Cervical Cancer in Their Future*.

[11] Bruni L., Diaz M., Barrionuevo-Rosas L. (2016). Global estimates of human papillomavirus vaccination coverage by region and income level: a pooled analysis. *The Lancet Global Health*.

[12] Ministero della Salute (2019). *National Vaccination Plan 2017-2019*.

[13] Ministero della Salute (2019). *HPV Vaccination coverages in Italy in 2017*.

[14] Jean S., Elshafei M., Buttenheim A. (2018). Social determinants of community-level human papillomavirus vaccination coverage in aschool-based vaccination programme. *Sexually Transmitted Infections*.

[15] Michail G., Smaili M., Vozikis A., Jelastopulu E., Adonakis G., Poulas K. (2014). Female students receiving post-secondary education in Greece: the results of a collaborative human papillomavirus knowledge survey. *Public Health*.

[16] Palmeri S., Costantino C., D'Angelo C. (2017). HPV vaccine hesitancy among parents of female adolescents: a pre–post interventional study. *Public Health*.

[17] Restivo V., Costantino C., Fazio T. (2018). Factors associated with HPV vaccine refusal among young adult women after ten years of vaccine implementation. *International Journal of Environmental Research and Public Health*.

[18] Tull F., Borg K., Knott C. (2019). Short message service reminders to parents for increasing adolescent human papillomavirus vaccination rates in a secondary school vaccine program: a randomized control trial. *Journal of Adolescent Health*.

[19] Coley S., Hoefer D., Rausch-Phung E. (2018). A population‐based reminder intervention to improve human papillomavirus vaccination rates among adolescents at routine vaccination age. *Vaccine*.

[20] Henrikson N. B., Zhu W., Baba L. (2018). Outreach and reminders to improve human papillomavirus vaccination in an integrated primary care system. *Clinical Pediatrics*.

[21] Bae J., Ford E. W., Wu S., Huerta T. (2017). Electronic reminder's role in promoting human papillomavirus vaccine use. *The American Journal of Managed Care*.

[22] Francis D. B., Cates J. R., Wagner K. P., Zola T., Fitter J. E., Coyne-Beasley T. (2017). Communication technologies to improve HPV vaccination initiation and completion: A systematic review. *Patient Education and Counseling*.

[23] Rand C. M., Vincelli P., Goldstein N. P., Blumkin A., Szilagyi P. G. (2017). Effects of phone and text message reminders on completion of the human papillomavirus vaccine series. *Journal of Adolescent Health*.

[24] Das J. K., Salam R. A., Arshad A., Lassi Z. S., Bhutta Z. A. (2016). Systematic review and meta-analysis of interventions to improve access and coverage of adolescent immunizations. *Journal of Adolescent Health*.

[25] CDC (2019). *Immunization Strategies for Healthcare Practices and Providers*.

[26] Lehmann C. E., Brady R. C., Battley R. O., Huggins J. L. (2016). Adolescent vaccination strategies: interventions to increase coverage. *Pediatric Drugs*.

[27] Hardt K., Bonanni P., King S. (2016). Vaccine strategies: Optimising outcomes. *Vaccine*.

[28] The Community Preventive Services Task Force (CPSTF) (2019). *The Community Guide*.

[29] CPSTF Findings for Increasing Vaccination (2019). *The Community Guide*.

[30] Amdisen L., Kristensen M. L., Rytter D., Mølbak K., Valentiner-Branth P. (2018). Identification of determinants associated with uptake of the first dose of the human papillomavirus vaccine in Denmark. *Vaccine*.

[31] Albright K., Barnard J., O'Leary S. T. (2017). Noninitiation and noncompletion of hpv vaccine among english- and spanish-speaking parents of adolescent girls: a qualitative study. *Academic Pediatrics*.

[32] Ko L. K., Taylor V. M., Mohamed F. B. (2019). “We brought our culture here with us”: A qualitative study of perceptions of HPV vaccine and vaccine uptake among East African immigrant mothers. *Papillomavirus Research*.

[33] Flores Y. N., Salmerón J., Glenn B. A., Lang C. M., Chang L. C., Bastani R. (2019). Clinician offering is a key factor associated with HPV vaccine uptake among Mexican mothers in the USA and Mexico: a cross-sectional study. *International Journal of Public Health*.

[34] Balogun F., Omotade O., Maree J. (2018). “She must have been sleeping around”…: Contextual interpretations of cervical cancer and views regarding HPV vaccination for adolescents in selected communities in Ibadan, Nigeria. *PLoS ONE*.

[35] Painter J. E., Viana De O. Mesquita S., Jimenez L., Avila A. A., Sutter C. J., Sutter R. (2018). Vaccine-related attitudes and decision-making among uninsured, Latin American immigrant mothers of adolescent daughters: a qualitative study. *Human Vaccines & Immunotherapeutics*.

[36] Abou El Ola M., Rajab M., Abdallah D. (2018). Low rate of human papillomavirus vaccination among schoolgirls in Lebanon: barriers to vaccination with a focus on mothers&rsquo; knowledge about available vaccines. *Therapeutics and Clinical Risk Management*.

[37] Wang L. D., Lam W. W., Fielding R. (2017). Determinants of human papillomavirus vaccination uptake among adolescent girls: A theory-based longitudinal study among Hong Kong Chinese parents. *Preventive Medicine*.

[38] Pot M., van Keulen H. M., Ruiter R. A., Eekhout I., Mollema L., Paulussen T. W. (2017). Motivational and contextual determinants of HPV-vaccination uptake: A longitudinal study among mothers of girls invited for the HPV-vaccination. *Preventive Medicine*.

[39] Schülein S., Taylor K. J., König J., Claus M., Blettner M., Klug S. J. (2016). Factors influencing uptake of HPV vaccination among girls in Germany. *BMC Public Health*.

[40] Tung I. L., Machalek D. A., Garland S. M., Consolaro M. E. (2016). Attitudes, knowledge and factors associated with human papillomavirus (HPV) vaccine uptake in adolescent girls and young women in victoria, Australia. *PLoS ONE*.

[41] Wilson A. R., Hashibe M., Bodson J. (2016). Factors related to HPV vaccine uptake and 3-dose completion among women in a low vaccination region of the USA: an observational study. *BMC Women's Health*.

[42] Holloway G. L. (2019). Effective HPV Vaccination Strategies: What Does the Evidence Say? An Integrated Literature Review. *Journal of Pediatric Nursing*.

[43] Oliver K., Frawley A., Garland E. (2016). HPV vaccination: Population approaches for improving rates. *Human Vaccines & Immunotherapeutics*.

[44] Perkins R. B., Chigurupati N. L., Apte G. (2016). Why don't adolescents finish the HPV vaccine series? A qualitative study of parents and providers. *Human Vaccines & Immunotherapeutics*.

[45] Jacobson R. M., Agunwamba A. A., St. Sauver J. L., Finney Rutten L. J. (2015). The most effective and promising population health strategies to advance human papillomavirus vaccination. *Expert Review of Vaccines*.

[46] McLean H. Q., VanWormer J. J., Chow B. D. (2017). Improving Human Papillomavirus Vaccine Use in an Integrated Health System: Impact of a Provider and Staff Intervention. *Journal of Adolescent Health*.

[47] Ministry of Health and Istituto Superiore di Sanità (2019). *Local and evaluation of HPV immunization campaigns against HPV, VALORE*.

